# The Harrison Anti-Narcotic Law

**Published:** 1915-08

**Authors:** Hooper Alexander

**Affiliations:** U. S. District Attorney for the Northern District of Georgia, Atlanta, Georgia


### THE HARRISON ANTI-NARCOTIC LAW.

By Mr. Hooper Alexander, U. S. District Attorney for the Northern District of Georgia, Atlanta, Georgia.

The Harrison Anti-Narcotic Law does not seek to interfere with the bona fide discretion of a physician in the legitimate practice of medicine. It leaves to his judgment, when honestly exercised, the sole determination of what amount of morphine is needed by a patient, if any.

It is a revenue measure and in prescribing regulations for the collection of revenue it requires that all sales of morphine be made on certain forms or on bona fide prescriptions.

It makes it unlawful for any person who is not registered under the law to have morphine in his possession, unless he is an employe of a registered vendor of morphine, a physician, or some person who obtained it on a prescription given in good faith.

In the efforts to carry out this law the Government officers have met considerable success, and incidentally it has resulted in a very considerable reduction in the sales.

As a result of this reduction there has been among the addicts some suffering, and the probabilities are that there will be more. It has seemed to the local officers here that it would be wise if some organized provision can be made to prevent or relieve this suffering during the temporary period which must follow the beginning of the enforcement of the law. This necessity is the explanation of why the Government officers have sought a conference with the medical profession of the city for the purpose of bringing existing conditions to your attention.

The Government officers are not undertaking to practice medicine nor to interfere with the practice of medicine. They can and will enforce the law and they feel entire confidence in the belief that they have the sympathy and will have the co-operation of your great and learned profession. It is confidently believed from evidence now in hand that decided progress has been made and that more progress will be made, as an incident to the law, in breaking up the practice of morphine eating or hypodermic injections by that large number of persons who have become addicts to morphine merely from the desire to use it and who do not need it. The statistical information in the hands of the revenue officers leads to the estimate that there are something like two thousand people in Atlanta regularly using morphine, and who have no need for it except as that need arises from mere habit.

The officers do not profess to understand the medical problems that are connected with this matter; they merely know that the number is very large, that considerable suffering is beginning to be manifest, and that the economic welfare of this city is suffering by reason of the fact that the use of morphine is destroying the energies of a great number of people, among whom are many competent young men, and that nearly all these profess at least a desire to be rid of the habit.

For this reason this conference has been sought with a view to laying the facts before your profession and ascertaining whether it is not possible for you to induce the city authorities to co-operate in making some provision for the relief of suffering. It is not the purpose of the officers to lay down any particular plan or even suggest one; they feel that that is a matter that your profession alone can determine. It has been suggested that it may be feasible for the regular practitioners of medicine to withhold individual prescriptions from the addicts and allow the city to provide for dispensing or prescribing through designated physicians to such extent as may be absolutely necessary for the prevention of suffering. Some objections have been suggested to this plan by individual physicians. What force may be in the suggestion or what force may be in the objections, in the nature of things, can not be known to the revenue officers or the legal officers of the Government. None but experienced men like the medical profession can determine those questions.

If some such plan should commend itself to your wisdom, it might be perhaps necessary to except those cases in which physicians have in their regular practice patients for whom they have prescribed in the past, and for whom they would continue to prescribe. It may be that in the family practice of individual physicians there will be found occasionally members of families for whom the individual physicians would prefer to continue to prescribe.

At the same time it is quite certain that there is a large number of these addicts who have no regular physician, and who have heretofore bought morphine at will from any drug store that would sell it, or from peddlers, or have procured it from such few physicians in the city as may desire to prescribe simply and solely as a means of making profit. This class is being very largely cut off from the supply heretofore open to them, and in some instances have suffered from the deprivation.

The officers of the Government have proceeded with all possible discretion and have endeavored to move with reasonable caution. They have discovered that there are some few physicians in the city whose practice it is to write prescriptions for all comers, according to their desires. It is the view of the Government that these prescriptions are not given in good faith; that the persons who obtain possession of morphine in this way do not obtain it upon prescriptions given in good faith, and are, therefore, liable under the eighth section of the Act. In such cases it is the further view of the Government, and I think there can be no doubt about this as a legal proposition, that the physician who gives the prescription in bad faith is equally guilty with the patient. The Government has no doubt of its ability to deal with this class of physicians; they will be prosecuted, and I think they will be convicted.

As I have already said, there is no doubt as to the ability and intention of the Government to enforce the law. It will enforce it. The object of this conference so far as the Government officials are concerned is not to request aid in the enforcement of the law, but to request aid in mitigating the inconvenience and suffering which may result from that rigid enforcement upon which the Government is definitely resolved.

With this statement of the matter, it is the desire of the Government officials to leave the subject to your body and to its wisdom to devise a plan which will be consistent with the law, and which at the same time will provide for the immediate needs of the addicts, and, we hope, for the rapid reduction and final elimination of the habit among those addicts who are otherwise bound either to continue the practice or suffer from its sudden cessation.

I shall be very glad to give any information that it may be in my power to give to those who may desire to make inquiry on the subject.

